# The Role of De Novo Variants in Formation of Human Anorectal Malformations

**DOI:** 10.3390/genes12091298

**Published:** 2021-08-24

**Authors:** Gabriel C. Dworschak, Iris A. L. M. van Rooij, Heiko M. Reutter

**Affiliations:** 1 Department of Pediatrics, Clinic for Pediatrics, University Hospital Bonn, 53127 Bonn, Germany; 2 Institute of Human Genetics, University Hospital Bonn, 53127 Bonn, Germany; 3 Institute for Anatomy and Cell Biology, University Hospital Bonn, University of Bonn, 53115 Bonn, Germany; 4 Department for Health Evidence, Radboud Institute for Health Sciences, Radboud University Medical Center (Radboudumc), 6525 EZ Nijmegen, The Netherlands; 5 Department of Neonatology and Pediatric Intensive Care, University Hospital Erlangen, 91054 Erlangen, Germany

**Keywords:** anorectal malformation (ARM), de novo, heritability, fecundity, copy number variation (CNV), birth defect

## Abstract

Anorectal malformations (ARM) represent a rare birth defect of the hindgut that occur in approximately 1 in 3000 live births. Around 60% of ARM occur with associated anomalies including defined genetic syndromes and associations with chromosomal aberrations. The etiology of ARM is heterogeneous, with the individual environmental or genetic risk factors remaining unknown for the majority of cases. The occurrence of familial ARM and previous epidemiologic analysis suggest autosomal dominant inheritance in a substantial subset of ARM patients. The implicated mortality and reduced fecundity in patients with ARM would lead to allele loss. However, mutational de novo events among the affected individuals could compensate for the evolutionary pressure. With the implementation of exome sequencing, array-based molecular karyotyping and family-based rare variant analyses, the technologies are available to identify the respective factors. This review discusses the identification of disease-causing variants among individuals with ARM. It highlights the role of mutational de novo events.

## 1. Introduction

Anorectal malformations (ARM) comprise a broad spectrum of birth defects, ranging from mild anal anomalies to complex cloacal malformations. The estimated birth prevalence is 1 in 3000 live births, with a male to female ratio of 1.7 [1,2,3,4]. Associated anomalies occur within approximately 60% of patients, most commonly involving the genitourinary tract, cardiovascular system, central nervous system and the skeletal system [4,5]. ARM may present as a feature of a defined genetic syndrome or in association with chromosomal aberrations [6,7]. In this respect, ARM may present non-syndromic (isolated) or syndromic (non-isolated). According to the case classification guidelines for the National Birth Defects Prevention Study [8], ARM patients with a chromosomal or single gene disorder, a defined clinical syndrome, mental retardation, and/or dysmorphisms have syndromic ARM. The clinical management of ARM is mainly reconstructive surgery and life-long symptomatic treatment (i.e., management of chronic constipation, incontinence, recurrent infections, and psychosocial support).

## 2. Established Genetic Factors in the etiology of ARM

**Figure 1:**
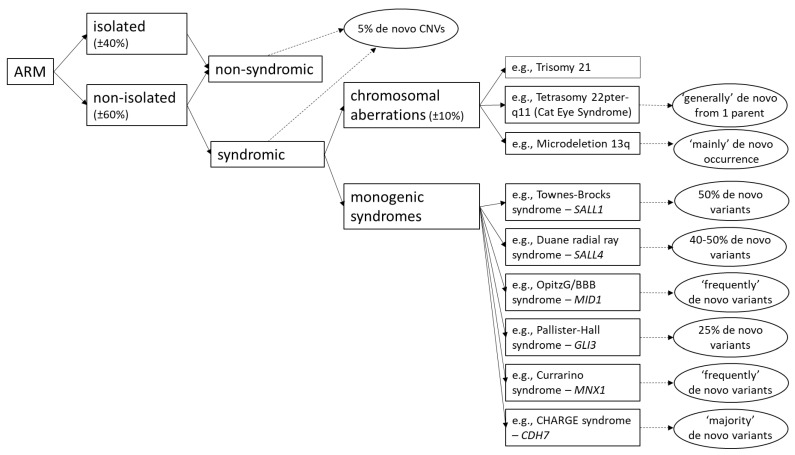
An overview of a selection of relevant genetic causes of ARM and the proportion of de novo variation.

Up to 10% of syndromic ARM are associated with numeric or structural chromosomal anomalies [5,9]. While for the majority of the remaining syndromic phenotypes that cause ARM remain elusive, about 30 known monogenic syndromes have been described with ARM as an associated phenotypic feature. Here, we discuss a selection of genetically defined syndromes with special emphasis on de novo variation (Figure 1).

### 2.1. Monogenic Syndromes

#### 2.1.1. Townes–Brocks Syndrome

Townes–Brocks syndrome (TBS; OMIM #107480) is characterized by ARM, thumb anomalies, renal anomalies, cardiac anomalies, dysplastic ears and hearing loss. TBS results from dominant variants in *SALL1* that occur in 50% of patients de novo [10]. Interestingly, pathogenic de novo *SALL1* variants most commonly affect the paternally derived chromosome (87.5%) without an obvious age effect [11]. In 2017, Webb et al. identified a *DACT1* variant in a three-generation family with features overlapping with TBS, negative for variants in *SALL1* [12]. In a re-sequencing study of 78 patients with ARM, no pathogenic *DACT1* variants were discovered [13] and no additional patient with a *DACT1* variant and a phenotype overlapping TBS has been reported since.

#### 2.1.2. Duane-Radial Ray Syndrome

The Duane-radial ray syndrome (OMIM #607323) is an autosomal dominant disorder characterized by upper limb, ocular, and renal anomalies caused by variants in *SALL4*. Less common features comprise sensorineural hearing loss and gastrointestinal anomalies, such as ARM. Pathogenic variants in *SALL4* occur in 40%–50% de novo [14].

#### 2.1.3. Opitz G/BBB Syndrome

The X-linked recessive Opitz G/BBB syndrome (OMIM #300000) is characterized by laryngotracheoesophageal abnormalities, hypospadias, hypertelorism and less commonly ARM, cardiac anomalies and syndactyly. Hemizygous pathogenic variants in *MID1* have been identified as the underlying genetic cause. Despite the Opitz G/BBB syndrome following an X-linked-recessive heritability, de novo events have been frequently reported [15,16,17].

#### 2.1.4. Pallister–Hall Syndrome

Pallister–Hall syndrome (OMIM #146510) is characterized by hypothalamic hamartoma, polydactyly, bifid epiglottis, ARM, and genitourinary tract anomalies. Heterozygous variants in *GLI3* have been described as causative and about 25% of patients have a de novo pathogenic variant [18,19]. Interestingly, patients with a de novo *GLI3* pathogenic variant are often more severely affected than patients with a family history of Pallister–Hall syndrome [20].

#### 2.1.5. Currarino Syndrome

Currarino syndrome (CS; OMIM #176450) is characterized by the triad of a presacral mass, sacral anomalies and ARM [21]. Heterozygous variants in *MNX1* have been identified in 92% of familial and 32% of sporadic cases [22]. The fraction of de novo *MNX1* variants has not been systematically studied, but there are frequent reports of de novo occurrence [23,24,25,26]. Since CS presents with variable expressivity and pathogenic variants may have a reduced penetrance, it is not surprising that completely asymptomatic individuals with pathogenic *MNX1* variants have been reported [27]. However, even if a patient appears to represent a sporadic case, screening of the parents for features of CS and genetic testing of the parents in the case of identification of a *MNX1* variant in the patient is recommended [22].

#### 2.1.6. CHARGE Syndrome

The CHARGE syndrome (#214800) comprises coloboma, heart defect, choanal atresia, growth retardation, developmental delay, genital hypoplasia, ear anomalies (including deafness) and ARM. Heterozygous variants in *CHD7* have been identified as causative. Pathogenic variants in *CHD7* occur in the majority of cases de novo [28] and affect predominantly the paternal allele [29]. About 70% of these variants represent nonsense or frameshift variants [30].

### 2.2. Chromosomal Anomalies

#### 2.2.1. Trisomy 21

Between 2% and 5% of patients with ARM have trisomy 21 [5,31] and vice versa about 0.9% of patients with Down syndrome present with ARM [32]. Over 95% of patients with Down syndrome and ARM have a rare form, i.e., imperforate anus without fistula [33]. The majority of patients with trisomy 21 have three free copies of chromosome 21 (95%); in the remaining patients, one copy is translocated to another acrocentric chromosome, most commonly chromosome 14 or 21 [34].

#### 2.2.2. Cat Eye Syndrome

Cat eye syndrome (OMIM #115470) comprises ocular colobomas, preauricular abnormalities, ARM and mild to moderate intellectual disability. Cat eye syndrome is caused by a partial tetrasomy (i.e., four copies) of the region spanning the p-arm and a part of 22q11 [35]. The extra material is usually in the form of a small supernumerary chromosome, frequently has two centromeres, is bisatellited, and represents an inv dup(22)(q11). The supernumerary chromosome 22 generally originates de novo from one of the parents [36].

#### 2.2.3. Microdeletion 13q

The phenotypic spectrum of 13q deletions is broad and comprises intellectual disability, growth retardation, renal, heart and brain malformation, ARM and other gastrointestinal abnormalities, genital abnormalities and limb malformation, especially absent or hypoplastic thumbs, and characteristic craniofacial dysmorphisms [37]. Interestingly, certain anomalies have been mapped to specific deleted regions [38]. The critical region for ARM has been described as 11 Mb, flanked by q33.1 (103 Mb) and q34 (qter) [38,39] and later refined to 13q33.3-qter [40]. While the ratio of de novo occurrence has not been systematically assessed, the reported series of patients suggest a de novo occurrence as the main cause for partial 13q deletions [40,41].

## 3. Candidate Genes and Copy Number Variants

The aforementioned syndromes with identified genetic causes, the occurrence of familial ARM and the finding of ARM in knockout mouse models strongly indicate a genetic contribution in the etiology of ARM. However, by the investigation of genes known to cause syndromes that share ARM as one of the features, only a small proportion of patients are molecularly solved [42]. Therefore, it appeared reasonable to conduct systematic genetic studies in order to explain the missing heritability.

Several studies have systematically employed array-based molecular karyotyping in order to investigate the genetic factors underlying ARM. In a cohort of 224 non-syndromic and syndromic ARM patients [40,43,44,45,46,47,48], 12 patients (5%) were identified with a pathogenic de novo CNV. Furthermore, the authors detected potentially pathogenic CNVs in six patients with unclear segregation since the respective parent(s) were not available for testing. Among the de novo CNVs two deletions comprised chromosomal region 13q33 [40]. Furthermore, four CNVs comprising chromosomal region 22q11.2 were identified. Here, one deletion and one duplication of chromosomal region 22q11.21 were confirmed to be de novo [43,46], and for two deletions only one parent was available for segregation testing [46,48]. Among these CNVs there were regions that have been repeatedly associated with ARM, such as chromosomal regions 22q11.21 and 13q33.

Another study investigating 363 ARM patients showed a 1.3-fold significant excess of rare CNVs in patients compared to controls [49]. In total, twelve chromosomal aberrations and 114 rare CNVs were detected in patients. However, these data are not comparable with the aforementioned studies, since the filtering of these variants was performed regardless of the inheritance pattern and segregation status.

A study of 123 patients with VACTERL-association (OMIM%192350) has applied a targeted re-sequencing approach of ciliary candidate genes as well as disease-associated genes. Heterozygous variants in *FOXF1* were previously reported in patients with alveolar capillary dysplasia and misalignment of the pulmonary veins [50]. The authors identified a de novo variant in *FOXF1* (p.Gly220Cys) in a patient with ARM, left-sided renal agenesis, and glandular hypospadias [51].

Another candidate re-sequencing study of 211 VACTERL and 204 ARM patients included 30 candidate genes that were described previously in relation to VACTERL features, either in animal models or in individual patients [42]. However, the authors did not identify loss-of-function variants in the candidate genes, indicating genetic heterogeneity in the etiology of ARM.

## 4. Epidemiological Aspects—A De Novo Paradigm

Although ARM is usually sporadic, the occurrence of familial ARM affecting multiple generations suggested autosomal dominant inheritance in, at least, a subset of families [52]. In an epidemiological study in a cohort of 1606 ARM patients Falcone et al. reported an additional family member with ARM in 1.4% of patients [53]. Later, in a study with 327 ARM patients the risk of recurrence between siblings was calculated, with 1% supporting the figure from Falcone et al. [54]. However, the same study suggested a recurrence risk of ARM of approximately one in two live births (62%) for parent–offspring transmission. This finding supports the hypothesis of autosomal dominant inheritance for a subset of ARM patients.

Not even five decades ago, ARM have been associated with a significant mortality and morbidity. Especially the implicated mortality and the reduced fecundity in patients with ARM lead to allele loss. Although the evolutionary pressure would eliminate such deleterious alleles, the prevalence of ARM has been relatively stable between 1980 and 2019 according to data of the European Surveillance of Congenital Malformations (EUROCAT) network (www.eurocat-network.eu, accessed on 28 July 2021) [55]. Since the human per-generation mutation rate is exceptionally high compared to other species, with an average newborn acquiring a total of 50 to 100 de novo variants [56], these variants may have severe phenotypic effects when they affect functionally important bases in the genome. The de novo occurrence of deleterious variants may explain a stable prevalence of disease in the human population. This paradigm is especially appropriate when the mutational target is large and includes many genes. Similar mechanisms have been shown for other disorders that compromise individual fecundity, such as mental retardation [57].

Due to the improvement of delicate surgical techniques, such as the definitive repair of ARM, sexual function can be preserved more often, resulting in more offspring of patients with ARM. This would lead to a higher burden of deleterious variants and ultimately lead to an increase in the prevalence of ARM. However, it remains to be seen how these factors will develop in the future.

## 5. Conclusions and Outlook

Several lines of evidence show different genetic factors to be involved in the development of ARM. These factors are heterogeneous and include chromosomal aberrations, copy number variants and single nucleotide variants. De novo variants contribute substantially to the epidemiologic disease burden. Similar to what has been shown for other genetic conditions associated with reduced fecundity, de novo variants may compensate for allele loss in patients with ARM.

Exploration and characterization of the complete genome will ultimately identify regulatory genetic elements that might also contribute to the formation of ARM. The identification of these de novo variations within these regulatory elements might complement the missing heritability among cases with ARM.

## Data Availability

Not applicable.
